# Supplementary material for: Chromothripsis during telomere crisis is independent of NHEJ, and consistent with a replicative origin
Source: Genome Res. 2019 May;29(5):737–49. doi: 10.1101/gr.240705.118 (PMC6499312; doi:10.1101/gr.240705.118)
Supplement: Supplemental Material [file supp_gr.240705.118_Supplemental_file_1.zip › contigs/annotated_contigs/DB82/contig.2.DB82_length_520_mean_cov_6.05769230769.docx]

**DB82_length_520_mean_cov_6.05769230769**

```
AAGCTTCTATCCTTTTATTCTCTTATTACACTCTGGCACACTGGTCTTCTTTCAGTTTTTCAGGCACATCAAACTCCTCCCAAGCTTGG
 >chr20:40906794-40907080 - E=1e-160 p=1e-02
GGCCTTCGCTCATCTTGCCCTCTCTCTGAAATGCTTTGGCTACTATATATGTCTCCTTAATAGATAAATATCTTAACAGAGAAGTATCT

TTAAGATGGCCTCTTAAACCTTATGTCCTCAGAAGACCATCCTGACCTCTGTTTTAATGTATATCCCTAATTTTCCACTCTCACTAAAC

CCTGTACTTTTCCTTCATA|T|AATCTTAGAAACATTATATGTGAGGCACCCAGAAAAGTACCTGGCATGTCGTGGGTGCTTGATAAGT
                     >chr20:40899131-40899364 - E=1e-128
GGTGCCTCTTACTCTTGTTTCATTTTTTTTAAATCTATTTCAGGCCCTGTTTTGTTAAATCATGATGTTCTTTTGCTATTTAGTGTGAG

CTCTTCAGAGCCACAAGAATTGCAGTTTTGTTCACCGTGCTGCACGTTTTCATGTGTGTTTCTTTCTCTCCTTCTCT
```
